# Pseudo-cerebrospinal fluid rhinorrhea following endoscopic endonasal transsphenoidal surgery for PitNET: a case report and literature review

**DOI:** 10.3389/fsurg.2026.1945557

**Published:** 2026-10-01

**Authors:** Guotao Peng, Jiankuai Zhou, Jun Mo

**Affiliations:** Department of Neurosurgery, The Fourth Affiliated Hospital of School of Medicine, and International School of Medicine, International Institutes of Medicine, Zhejiang University, Yiwu, China

**Keywords:** diagnosis, endoscopic endonasal surgery, management, pseudo-cerebrospinal fluid rhinorrhea, vasomotor rhinitis

## Abstract

Pseudo-cerebrospinal fluid (CSF) rhinorrhea, defined as non-CSF nasal discharge mimicking true CSF leakage, is a rare but clinically significant complication that may prompt unnecessary aggressive treatment. We report a case of a 64-year-old man who developed positional clear nasal discharge after endoscopic endonasal resection of recurrent pituitary neuroendocrine tumor (PitNET). Glucose oxidase testing was negative, and imaging demonstrated intact skull base reconstruction. Nasal endoscopy revealed bilateral crusting and purulent secretions without evidence of CSF leak. The condition was diagnosed as vasomotor rhinitis. Conservative management—including nasal debridement, antibiotic therapy, topical corticosteroids, and saline irrigation—led to complete symptom resolution. This case underscores the importance of differentiating pseudo-CSF rhinorrhea from true CSF rhinorrhea to avoid invasive interventions.

## Introduction

True CSF rhinorrhea is a common but important complication after endoscopic endonasal transsphenoidal surgery for pituitary tumor (1). For severe true CSF rhinorrhea, reoperation may need to repair the defect (2). Pseudo-cerebrospinal fluid rhinorrhea imitates similar symptoms as true CSF rhinorrhea but different pathophysiological mechanism and treatment (3). In the past, a small number of case reports have shown that pseudo-CSF rhinorrhea usually occurs after skull base surgery that required anatomy or resection of petrous or cavernous carotid, greater superficial petrosal nerve and pericervical sympathetic ganglionated plexus (4). Published reports describe pseudo-CSF rhinorrhea as nasal stuffiness and hypersecretion, sometimes accompanied by facial flushing; symptoms may be exacerbated by exertion or elevated ambient temperature, and ipsilateral lacrimation is typically absent (5). Some researchers suggest that this occurs due to the abnormal reinnervation of the accessory nasal mucosa autonomic nerve following injury to the greater superficial petrosal nerve or arterial peripheral nerve plexus (5, 6).

Inexperienced doctors may perform unnecessary surgical repair, leading to iatrogenic injury. Different from the previously reported cases, we report a case of pseudo-CSF rhinorrhea after endoscopic endonasal transsphenoidal surgery for PitNET. This case does not entail exposing the arterial peripheral nerve plexus, dissecting he greater superficial petrosal nerve, or addressing highly invasive neoplasms affecting the facial and trigeminal nerves.

Through the analysis of this case and a review of the existing literature, we aim to elucidate the potential etiology, the pathophysiological mechanisms underlying clinical manifestations, effective treatment approaches, and the differential diagnosis between Pseudo-CSF rhinorrhea and true cerebrospinal fluid leak, thereby assisting neurosurgeons in managing pseudo-cerebrospinal fluid leak more effectively.

## Case presentation

A 64-year-old male patient was found to have recurrent PitNET during hospital examination for decreased vision in the left eye. The patient's medical history included an 8-year history of hypertension, previous tibial surgical intervention 19 years prior, and a craniotomy for PitNET resection performed 4 years before the current presentation. Notably, the patient had no history of allergic rhinitis. Clinical examination revealed no significant abnormalities, and endocrine evaluation demonstrated normal pituitary function. Magnetic resonance imaging (MRI) revealed a lesion in the sellar region, radiologically consistent with recurrent PitNET (Figures 1A,B).

**Figure 1:**
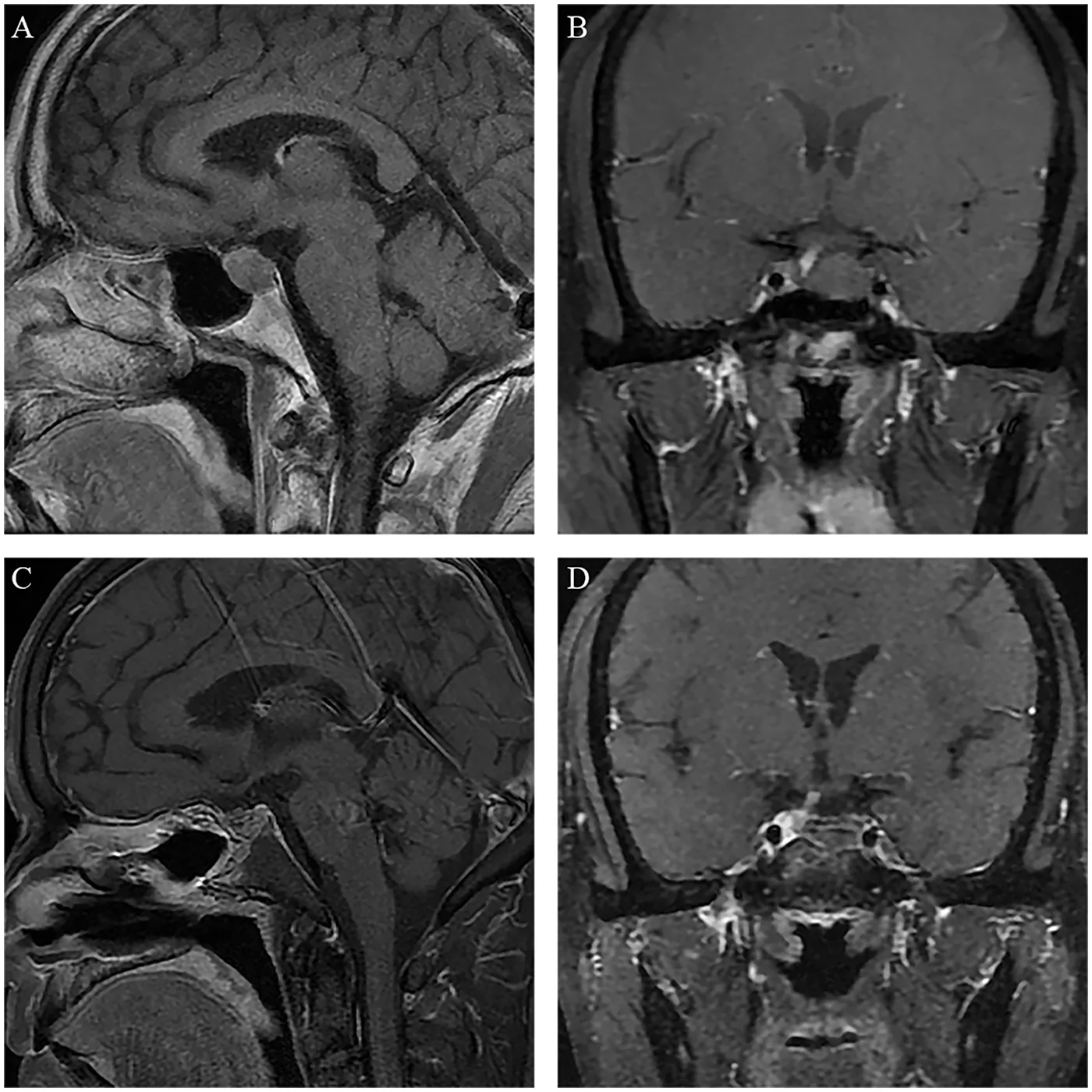
Magnetic resonance imaging (MRI) demonstrating pituitary neuroendocrine tumor (PitNET) before and after endoscopic endonasal transsphenoidal surgery. Preoperative **(A)** sagittal and **(B)** coronal views show the tumor location and extent. Postoperative **(C)** sagittal and **(D)** coronal views at 1-month follow-up demonstrate gross total resection and well skull base reconstruction.

The surgical intervention comprised an endoscopic endonasal transsphenoidal surgery for tumor resection performed via a bilateral nostril approach. Intraoperatively, a Grade 2 CSF leakage was encountered, necessitating skull base reconstruction utilizing a right nasoseptal flap (NSF). Subsequent histopathological analysis confirmed the diagnosis of PitNET. The patient's postoperative course progressed favourably, with no evidence of CSF leakage observed throughout the hospital stay. On sagittal MRI obtained on postoperative day 3, the surgical packing materials and the NSF were visible at the skull-base reconstruction site (Supplementary Figure S1). The patient discharged on postoperative day (POD) 11 with stable clinical parameters.

On POD 16, the patient presented with position-dependent left-sided nasal cavity fluid discharge upon forward head tilt that resolved in the supine position. There were no systemic symptoms such as fever and fatigue. Glucose oxidase testing of the nasal fluid yielded negative results, and nasal endoscopic examination performed with the patient seated and bending the head forward revealed no evidence of active CSF leakage. Conservative management, consisting of debridement of nasal secretions and surgical packing materials, resulted in successful resolution of the symptoms.

At one-month postoperative follow-up, the patient experienced recurrent episodes of clear fluid discharge from the left nasal cavity, notably exacerbated by the consumption of hot beverages and during meals (Figure 2A). Clinical evaluation revealed stable vital signs with absence of systemic manifestations such as fever or headache. Laboratory analysis of the nasal fluid yielded negative results on glucose oxidase testing. Contrast-enhanced MR demonstrated intact skull base reconstruction (Figures 1C,D). Nasal endoscopic examination revealed no evidence of true CSF rhinorrhea, the bilateral nasal cavities exhibited dry crusting and purulent discharge, predominantly affecting the right side, with an anterior septal perforation noted (Figure 2D). The coronal T2-weighted MRI showed no fluid accumulation within the sphenoid sinus and no definite imaging evidence of an active CSF leak (Figure 3).

**Figure 2:**
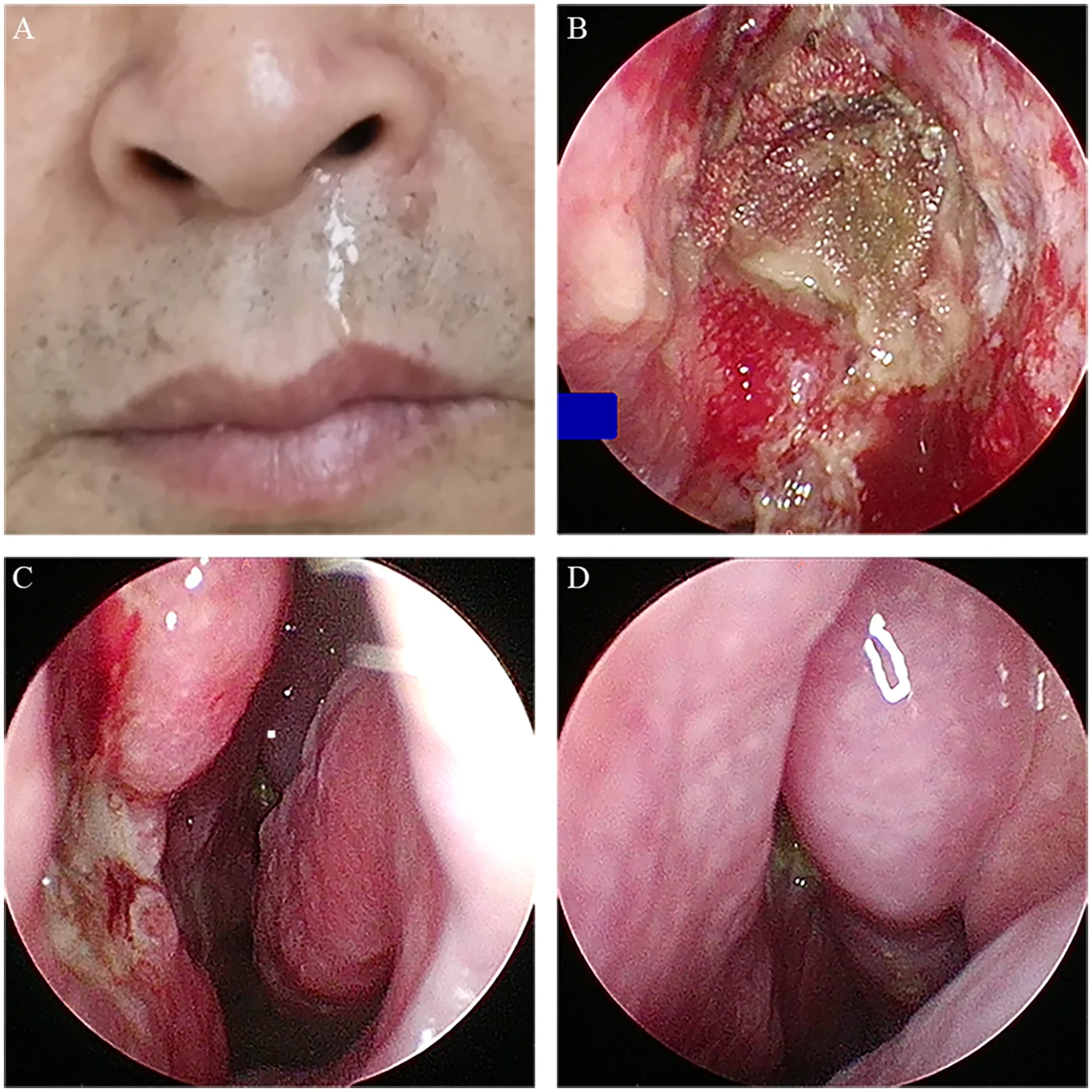
Endoscopic and clinical documentation of post-operative findings. **(A)** Left nasal cavity fluid discharge following hot beverage consumption at 1-month post-operative follow-up. **(B)** Right nasal cavity endoscopic examination on post-operative day 10. **(C,D)** Left nasal endoscopic examination during hospital admission at 1-month follow-up for evaluation of nasal discharge.

**Figure 3:**
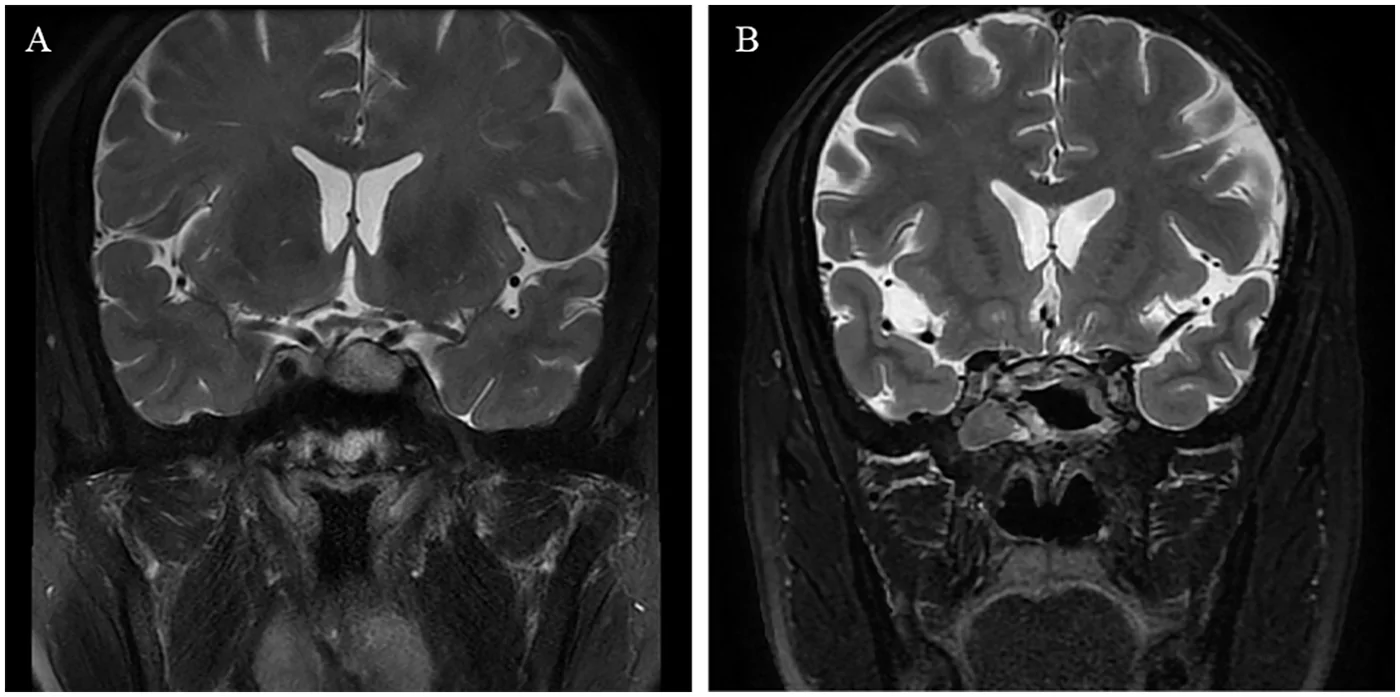
Preoperative and 1-month postoperative coronal T2-weighted magnetic resonance images. **(A)** Preoperative coronal T2-weighted image. **(B)** Coronal T2-weighted image obtained at the 1-month postoperative follow-up, showing no fluid accumulation within the sphenoid sinus or other definite imaging evidence of an active CSF leak.

Based on the clinical presentation and examination findings, a diagnosis of vasomotor rhinitis was considered. The therapeutic approach encompassed comprehensive nasal cavity debridement, antimicrobial therapy with ceftriaxone, topical corticosteroid administration via budesonide nasal spray, and regular nasal irrigation utilizing saline solution at a frequency of 3–6 times daily. This multimodal treatment strategy proved efficacious, resulting in complete resolution of the patient's postprandial nasal discharge symptoms.

## Differential diagnosis

Accurate differentiation between true CSF rhinorrhea and pseudo-CSF rhinorrhea is crucial for determining appropriate therapeutic interventions, as t pseudo-CSF rhinorrhea necessitate surgical intervention while pseudo-CSF rhinorrhea typically requires conservative management. The clinical characteristics and diagnostic features of these conditions are summarized in Table 1. Pseudo-CSF rhinorrhea typically manifests within months to years post-surgery, with symptom exacerbation triggered by physical exertion, emotional stress, or elevated ambient temperature. A characteristic feature includes concurrent ipsilateral lacrimation reduction. In contrast, true CSF rhinorrhea demonstrates position-dependent aggravation, particularly during postural changes or Valsalva maneuver, without correlation to emotional state, dietary intake, or ambient temperature. Clinical presentation of pseudo-CSF rhinorrhea may include nasal mucosal congestion and hypersecretion, notably absent of meningeal irritation signs. Conversely, true CSF rhinorrhea may present with meningeal symptoms. Laboratory analysis reveals that *β*2-transferrin, a highly specific CSF biomarker, is characteristically negative in pseudo-CSF rhinorrhea but positive in true CSF rhinorrhea. In our institution, where *β*2-transferrin testing was unavailable, glucose oxidase testing served as an alternative diagnostic tool. Radiological findings also provide distinctive features: pseudo-CSF rhinorrhea typically demonstrates unremarkable CT and MRI studies without evidence of skull base defects or CSF leak, whereas true CSF rhinorrhea often reveals identifiable skull base disruption or CSF accumulation. Therapeutic response further aids differentiation, as pseudo-CSF rhinorrhea typically shows improvement with conservative measures including topical anticholinergics and corticosteroids, while true CSF rhinorrhea generally requires surgical intervention for definitive management.

**Table 1:** Differential diagnoisis of true CSF rhinorrhea and pseudo-CSF rhinorrhea.

Characteristic	True CSF rhinorrhea	Pseudo-CSF rhinorrhea
Occurrence time	Immediate onset after head trauma or surgery	Develops months or years after surgery
Aggravating factors	Worsens with head-down position or Valsalva maneuvers	Exacerbated by physical exertion, emotional stress, or elevated ambient temperature
Associated symptoms	May present with meningeal irritation signs (neck stiffness, meningitis)	Shows nasal mucosal congestion and increased secretions with ipsilateral tear reduction; no meningeal signs
*β*2 transferrin in nasal secretions	Positive	Negative
Glucose content in nasal secretions	Elevated	Negative
Imaging tests	May reveal skull base defects or CSF leak	Typically shows no evidence of skull base defects or CSF leak
Treatment	Often require surgical repair	Responds to intranasal ipratropium bromide and budesonide sprays

.

## Outcome and follow-up

At the 3-month follow-up, the patient was in good clinical condition with no recurrence of nasal discharge. He was advised to undergo nasal endoscopy in the future if nasal discharge recurs.

## Discussion

Pseudo-CSF rhinorrhea is usually associated with autonomic dysfunction, especially involving damage to the anterior parasympathetic nerve fibers that supply the sphenopalatine ganglion (7). In 1994, Cusimano et al. first reported 11 patients with pseudo-CSF rhinorrhea, who usually developed rhinorrhea after cranial base surgery involving the temporal bone or cavernous sinus carotid artery. CSF leakage was excluded by *β*2-transferrin detection (8). The sphenopalatine ganglion plays a key role in regulating the vasodilation and secretion function of the nasal mucosa and the sphenopalatine ganglion is located in the sphenopalatine fossa and is close to the posterior part of the upper medial angle of the maxillary sinus (6). Therefore, it is more susceptible to direct injury due to skull base trauma and/or surgery. In 2015, Grannan et al. reported a case of pseudo-CSF rhinorrhea in a 46-year-old man after transtemporal resection of a tumor in the cerebellopontine angle. The patient 's rhinorrhea is aggravated during chewing, which is different from previous pseudo-CSF rhinorrhea cases. Given these differing traits from the cases previously detailed, the author suggests a fresh theory that the cross-connection between the fifth cranial nerve and the facial nerve could be the reason for pseudo-CSF rhinorrhea in this individual (9).

This report presents distinct features from previous reports, involving a patient who underwent endoscopic endonasal transsphenoidal surgery for recurrent PitNET without evidence of nerve injury typically associated with pseudo-CSF rhinorrhea. Although initial concerns of CSF leakage arose due to postoperative nasal discharge and intraoperative CSF leakage, several clinical findings contradicted this diagnosis: the discharge was exclusively triggered by hot beverage consumption, glucose testing of the nasal fluid was negative, and postoperative MRI demonstrated intact nasoseptal flap reconstruction. Furthermore, the significant improvement in symptoms following budesonide nasal spray administration suggested an alternative diagnosis. We propose that postoperative vasomotor rhinitis following anterior skull base surgery may represent an underrecognized yet significant mechanism underlying pseudo-CSF rhinorrhea after endoscopic endonasal neurosurgery.

VMR represents a common non-allergic rhinitis characterized by persistent or intermittent nasal manifestations, including congestion, runny nose, and sneezing (10). Environmental triggers, particularly thermal and humidity fluctuation, can precipitate symptomatic episodes, notably evidenced by rhinorrhea following hot beverage consumption (Figure 2A). The pathophysiological mechanism underlying VMR primarily involves the dysregulation of neurovascular and endocrine regulation of the nasal mucosal vasculature and glandular function. While the pathogenesis of VMR remains incompletely elucidated, current studies have suggested critical involvement of various neuropeptides, such as substance P (SP), calcitonin gene-related peptide (CGRP), and vasoactive intestinal peptide (VIP) (11). Therapeutic management predominant relies on intranasal glucocorticoids, such as budesonide and mometasone furoate, which can effectively relieve symptoms such as nasal congestion and runny nose (12).

During endoscopic endonasal surgical procedures, the intranasal manipulation can potentially result in injury to both the nasal mucosal tissue and adjacent neural structures, particularly the nasociliary branch of the trigeminal nerve, subsequently leading to alterations in nasal mucosal sensitivity and disruption of normal physiological responses. For example, some patients reported nasal dryness, nasal congestion and nasal foreign body sensation after surgery, which may be related to changes in local nerve sensitivity. Skull base reconstruction typically employs multilayer repair techniques, incorporating various materials such as autologous fascia, allogeneic grafts, or NSF, each with distinct physiological implications for the nasal mucosa. While NSF effectively prevents CSF leakage by covering skull base defects, this surgical modification possibly alter nasal aerodynamics, including airflow patterns and pressure gradients, potentially disrupting mucosal homeostasis and heightening local sensitivity (13, 14). Furthermore, the introduction of synthetic materials may elicit localized immunological responses, resulting in enhanced inflammatory mediator release and subsequent augmentation of mucosal sensitivity (15, 16). Surgical operation possibly has a great influence on the recovery of nasal mucociliary function. Ciliary function is essential for mucus transport and nasal self-cleaning. Surgery destroys the ciliary structure of the nasal mucosa, resulting in weakened cleaning function, making postoperative patients prone to nasal dryness, obstruction or infection, which in turn affects the sensitivity of the nasal mucosa. Some studies have shown that cilia function can be gradually restored through appropriate postoperative care and local moist treatment (17). Postoperative blood supply instability may cause nasal mucosal congestion or dryness and other symptoms (Figures 2C,D), thus affecting the overall sensitivity of the nasal cavity. The patient 's nasal discharge was improved after nasal endoscopy or nasal cleaning, which indirectly confirmed the effect of nasal inflammatory factors on nasal discharge. Some antihypertensive drugs may affect the normal regulation of nasal vasoconstriction. *β*-blockers competitively inhibit *β*-adrenergic receptors on nasal mucosa, resulting in decreased cAMP production, smooth muscle contraction is enhanced, making the nasal mucosa more sensitive and causing congestion and nasal congestion (18). Calcium channel blockers are commonly used to treat hypertension and heart disease, and may also affect nasal blood flow and mucosal function. Calcium channel blockers have been studied for cold-induced nasal vasodilation, especially in controlling nasal blood flow and vascular response (19). This suggests that calcium channel blockers may have a direct effect on nasal blood vessels, thereby affecting the physiological state of the nasal mucosa.

The identification of true and false CSF rhinorrhea is very important. The complication of pseudo-CSF rhinorrhea is easy to occur after nasohypophysial surgery, and true CSF rhinorrhea is more inclined to be considered after nasal discharge. If pseudo-CSF rhinorrhea is misdiagnosed as true cerebrospinal fluid rhinorrhea, the patient may undergo some invasive tests and procedures, such as lumbar puncture, re-repair of cerebrospinal fluid leakage, which carry their own risks, including infection, bleeding, nerve damage and so on. Cusimano et al. reported 11 patients with nasal discharge after skull base surgery, none of whom had CT findings consistent with CSF leakage (5). This had led to some patients receiving unsuccessful attempts at repair. In the present case, VSM can be considered as a potential cause of pseudo-CSF rhinorrhea, and steroid drugs such as fluticasone propionate, beclomethasone, and budesonide nasal spray can be used to reduce nasal mucosal secretion (12). A complete nasal wash with saline solution may also help to relieve the symptoms of excessive nasal discharge before attempting a steroid nasal spray. Patients may also be associated with rhinitis, and antibiotics can be given if bacteria are detected after nasal fluid sampling and culture. The conservative management of pseudo-CSF rhinorrhea is a gradual and systematic process, wherein new therapeutic approaches are incrementally introduced, ensuring that patient safety and well-being are prioritized. Previous treatment of VSM suggests vidian neurectomy as a potential intervention strategy when drug intervention alone is ineffective (20). Without proper intervention, patients with pseudo-CSF rhinorrhea may develop into a persistent disease (3, 4). The condition of rhinorrhea may last for years and aggravate, and patients are at risk of developing rhinitis, sinusitis and other diseases (21). In our case, no rhinorrhea was observed during the follow-up period, indicating that active and correct early intervention of pseudo-CSF rhinorrhea will bring good prognosis to patients.

Several limitations of this case should be acknowledged. A dedicated MRI/CT CSF-rhinorrhea study protocol was not performed, which may have reduced the sensitivity for detecting a small, low-flow, intermittent, or position-dependent CSF leak. Nevertheless, the diagnosis was based on the concordance of the patient's clinical presentation, the absence of active leakage during position-provoked nasal endoscopy, the negative glucose oxidase test, and the marked response to conservative nasal treatment. Collectively, these findings allowed us to clinically exclude a persistent active CSF leak and supported postoperative pseudo-CSF rhinorrhea as the most likely diagnosis. Considering the low clinical probability of a persistent CSF leak and the favorable response to conservative treatment, further invasive or potentially unnecessary investigations were not pursued at that time. Dedicated protocol-specific imaging should be considered if suspicious nasal discharge recurs during follow-up.

## Conclusion

Pseudo-CSF rhinorrhea represents a rare but clinically significant postoperative complication following endoscopic endonasal transsphenoidal surgery. VMR deserves attention as a significant etiological mechanism in the pathogenesis of pseudo-CSF rhinorrhea. Prompt and accurate differential diagnosis is paramount for optimal patient management, as it prevents unnecessary surgical interventions and associated morbidity. In this case, conservative pharmacological treatment has demonstrated favorable outcomes.

## Data Availability

The original contributions presented in the study are included in the article/Supplementary Material, further inquiries can be directed to the corresponding author.

## Supplementary material

SUPPLEMENTARY FIGURE S1 Sagittal magnetic resonance image obtained on postoperative day 3 following endoscopic endonasal transsphenoidal surgery. The white arrow indicates the surgical packing materials consisting of gelatin sponge and a collagen plug, while the red arrow indicates the right-sided nasoseptal flap used for skull-base reconstruction.
